# Evaluation of field calibration methods and performance of AQMesh, a low-cost air quality monitor

**DOI:** 10.1007/s10661-021-09033-x

**Published:** 2021-04-08

**Authors:** Dan Wahlborg, Mikael Björling, Magnus Mattsson

**Affiliations:** 1 Department of Electrical Engineering, Mathematics and Science, Faculty of Engineering and Sustainable Development, University of Gävle, Gavle, Sweden; 2 Department of Building Engineering, Energy Systems and Sustainability Science, Faculty of Engineering and Sustainable Development, University of Gävle, Gavle, Sweden

**Keywords:** AQMesh, Bisquare linear fit, Orthogonal regression, Low-cost air quality monitor, Linear calibration

## Abstract

Field calibrations of NO_2_, NO, and PM_10_ from AQMesh Air Quality Monitors (AQMs) were conducted during a summer and an autumn period in a busy street in a midsize Swedish city. All the three linear calibration procedures studied (postscaled, bisquare, and orthogonal data) significantly reduced the ranges and magnitudes of the performance indicators to yield more reliable results than the raw data. The improvements were sufficient to satisfy the European Union (EU) Data Quality Objective (DQO) for indicative measurements as compared to reference data only for NO_2_ (above 50 µg m^−3^) and NO (above 30 µg m^−3^) during the autumn calibration period. The relatively simple bisquare procedure had the best performance overall. The bisquare procedure improved the root mean square error by the same amount as other studies using complex multivariate calibration methods. Low concentrations of pollutants were measured, far below the EU Environmental Quality Standard thresholds and even satisfying the future goals for the Environmental Quality Objectives. Cleaning the raw data by removing data points in the reference data that were below the reference station limit of detections (and the synchronous data points in the AQM prescaled data) was found to improve the performances of the calibration procedures appreciably. Many NO_2_ and almost all PM_10_ data points in this study fell below the AQM limit of detection. These low concentrations will probably be a common problem in many field studies, at least in areas with relatively low air pollution. However, the relative errors were sufficiently low for these data points that they could be interpreted as accurately representing low concentrations and did not need to be removed from the datasets. For the NO_2_ measurements, a slight periodic error correlated with sunlight and increased ambient temperature was noted. NO measurements correlated strongly with increased traffic.

**Supplementary information:**

The online version contains supplementary material available at 10.1007/s10661-021-09033-x.

## Introduction

Globally, 55% of the world population in 2018 (more than four billion people) resided in urban areas (United Nations, 2018). According to the World Health Organization, at least 80% (WHO, 2016) of this population is exposed to air pollutant concentrations exceeding the Air Quality Guidelines established with the goal of preserving human health and well-being (WHO, 2005).

Increased levels of airborne particulate matter (PM) are estimated to be solely responsible for approximately three million deaths annually, including nearly 170,000 children younger than 5 years old (WHO, 2016). In the European Union (EU), the levels of PM emissions (PM_10_ and PM_2.5_, i.e., PM with an aerodynamic diameter less than 10 and 2.5 μm respectively) have decreased since 2000 (European Environment Agency, 2019). Nevertheless, it has been shown that long-term exposure to low levels of airborne PM may cause cancer and cardiovascular and respiratory diseases (Hoek et al., 2013) and thereby raise mortality rates. Even short-term exposure to high levels of PM_10_ induces coughing in children with asthma and causes them to have difficulty breathing (Weinmayr et al., 2010).

The main sources of PM in urban environments in Sweden are wood combustion, road dust, and vehicle exhaust (SEPA, 2019). Emissions from wood combustion primarily originate from the use of small domestic stoves and fireplaces. Road dust particles are generated from wear debris from vehicle brakes and tires as well as road surface asphalt. The use of studded tires has been shown to significantly contribute to the latter problem, and there is also a clear connection between the use of studded tires and the amount of road dust (Swedish Environmental Research Institute, 2018).

In Sweden, more than 86% of the population is urban, and this fraction is expected to exceed 90% by 2030 (United Nations, 2018). At present, the urban population’s exposure to PM is comparatively low. According to the Swedish Environmental Protection Agency (SEPA), levels of PM in Swedish cities have been below the Environmental Quality Standards (EQS)-stipulated mean annual threshold (≤ 40 µg m^−3^) during the past 10 years (SEPA, 2019). The EQS-threshold is the legal limit that should not be exceeded. The present levels may also be compared to the Environmental Quality Objective (EQO) level for PM, which is the goal for how clean the air should be by 2030. In 2016, all large and many medium-sized cities exceeded the Swedish EQO mean annual (≤ 15 µg m^−3^) and daily (≤ 30 µg m^−3^) levels of PM_10_ exposure concentrations.

The concentration of nitrogen dioxide (NO_2_) in the air is strongly dependent on the level of traffic (Pleijel et al., 2004), and NO_2_ is commonly used as an air quality indicator. Animal studies have demonstrated that NO_2_ exposure causes numerous effects on several organs (WHO, 2005), and studies on lung cells in mice have shown that the lowest observed effective concentration of NO_2_ required for alteration at the cellular level is 0.2–0.4 ppm or 384–768 µg m^−3^, which corresponds to rush hour traffic emissions (WHO, 2000). Long-term exposure to NO_2_ in children aged 5–12 years has been reported to cause a 20% increase in the risk of respiratory disease for every increase in NO_2_ concentration of 28.3 µg m^−3^ (two-week average) (WHO, 2000). Evidence suggests that simultaneous exposure to NO_2_ and aeroallergens has a synergistic effect in inducing asthma in children (WHO, 2000). Evidence for the negative health effects of short-term exposure to NO_2_ is not as clear as that of short-term exposure to PM: some recent research on the short-term effects of NO_2_ on children with asthma indicates that NO_2_ has an adverse effect on their respiratory health, but no explicit conclusion has been drawn (Weinmayr et al., 2010).

Like NO_2_, nitrogen oxide (NO) primarily originates from combustion engine vehicle exhaust (Spindt et al., 1956), but it can also originate naturally from a photochemical reaction in which sunlight splits NO_2_ into NO and O. Sweden does not have an EQS level specified for NO by itself but includes NO in its mean annual concentration of 30 µg m^−3^ for mixed nitrogen oxides (NO_x_) in areas unaffected by cities or traffic (SEPA, 2019).

Monitoring air quality in populated areas is crucial for discovering pollution and preventing its negative effects on human health and the environment. According to the Air Quality Directive 2008/50/EC (EU, 2008), which applies to all EU member states, facilities for measuring air quality must be established in all urban areas. The reference stations employed should (requirements depend on the level of air pollution and city size) measure the concentrations of sulfur dioxide, NO_2_, NO, PM (PM_2.5_ and PM_10_), lead, benzene, and carbon monoxide according to rather strict criteria for measurement accuracy. These reference stations are now part of a global network reporting results to WHO and the EU. Currently, continual measurements of air quality are conducted in at least 3000 cities in 103 countries (WHO, 2016).

The comparatively high cost of reference stations (that meet the required measurement accuracy) limits the number of possible sampling points. However, more detailed mapping of air quality within urban areas is of increasing interest. One way to achieve increased mapping is to use low-cost multi-sensor platforms that are sufficiently accurate to indicate air quality in many different locations of a city. Easy, reliable access to data in real time from such air quality monitors (AQMs) could also be used to improve online city maps of the local air quality. Recent studies have proposed many small-sized and low-cost AQMs as candidates for these tasks (Popoola et al., 2018; Munir et al., 2019; Suriano et al., 2015; Borrego et al., 2016; Hamm et al., 2016).

Our study focuses on one of the aforementioned AQMs, namely, AQMesh (Castell et al., 2016, 2018; Hickman et al., 2017). Specifically, our objective is to evaluate the performance of calibration procedures for this AQM under field conditions. For this study, eight AQMesh AQMs were co-located with a reference station, and the technical performances of three calibration procedures were investigated.

## Methods

### Measurement devices

The AQMesh AQM (Environmental Instruments Ltd., UK, Gas algorithm v4.2.3 and PM algorithm v2.0) is an air quality multi-sensor platform capable of measuring particles of different size fractions with an optical particle counter, several gases with electrochemical (EC) gas sensors, temperature, and humidity (AQMesh, 2017). In this study, these AQMs were equipped with EC sensors (B4-series, Alphasense, UK) to measure NO, NO_2_, and ozone (O_3_). The PM_1_ (PM with diameter < 1 μm), PM_2.5_, and PM_10_ size-fractions are estimated by binning and converting AQM particle counts into PM size-based fractions on the assumptions of spherical particle shape and standard density. Only the measurements of the PM_10_ fraction will be used and compared to the reference station data in this paper. The AQMesh limits of detection (LODs) for NO, NO_2_, and PM_10_ were estimated by the manufacturer to be 6.25, 19.2, and 30 µg m^−3^, respectively (AQMesh, 2017). The individual sensors were factory-calibrated and mounted into the AQMs, and all AQMs were then co-located with the air quality reference station in Gävle. Data from the AQMs were uploaded via individual SIM cards and GPRS communication to a remote database. The transmitted raw data as directly obtained from the AQMs will be referred to as *prescaled data*.

The reference station, maintained by SLB-analys (Stockholms Luft- och Bulleranalys, Sweden) and approved by SEPA (SEPA, 2013), was equipped with a Chemiluminescent NO_x_ Analyser Model AC32M (Environnement SA, Poissy, France) for measuring nitric oxide (NO) and NO_2_ and an Ambient Particulate Monitor (TEOM® 1400AB, Thermo Scientific, Franklin, Massachusetts, USA) for measuring PM_10_. The LODs of the reference station NO, NO_2_, and PM_10_ were 3.375, 5.185, and 2.000 µg m^−3^, respectively (SEPA, 2013). The *reference data*, in which PM_10_ data were corrected for volatility (SEPA, 2013), were kindly provided by SLB-analys. The scope of this paper includes calibration of the AQMs by using the reference data. In this case, reference data were available for NO, NO_2_, and PM_10_.

### Field calibration

**Fig. 1:**
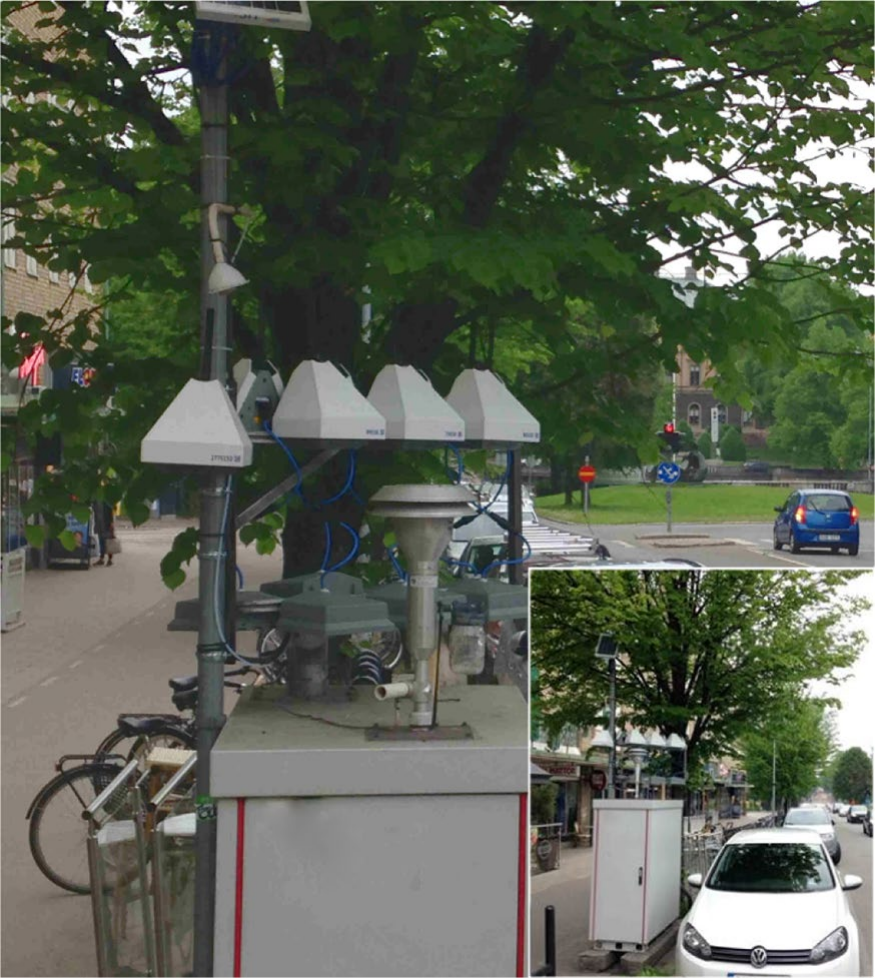
The experimental setup with 8 AQMs on top of a reference station

**Fig. 2:**
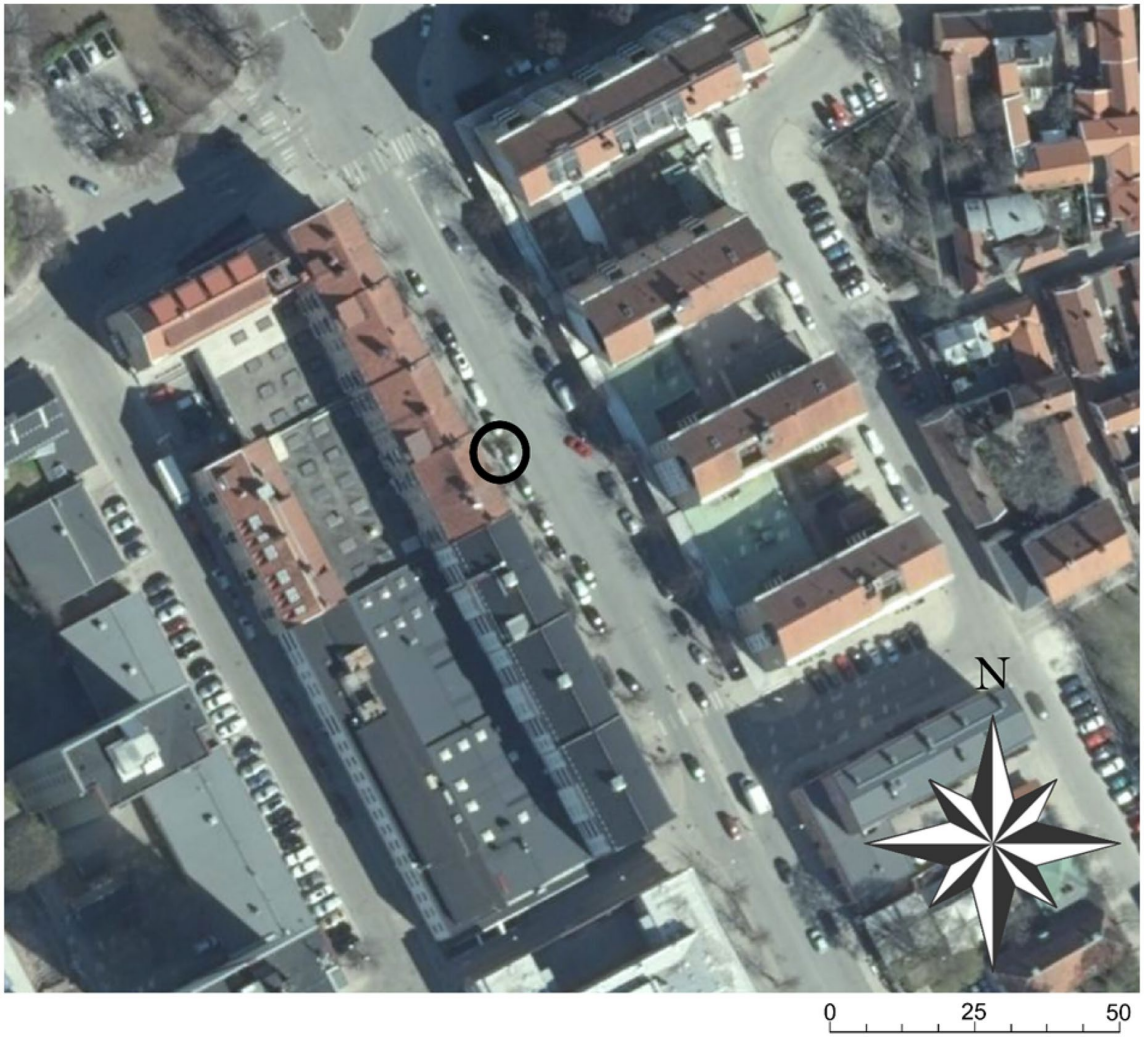
The measurement location on the west side of a busy street in Gävle, Sweden

Eight AQMs were mounted side by side at a height of 3m near the inlet of an air quality reference station (Fig. 1) located on the west side of a busy street in Gävle, Sweden. One of the eight AQMs had a solar panel power supply. The other seven AQMs had battery packs. The measurement interval was 15 min, and a battery pack lifetime of 7 months was expected. The street is approximately 25 m wide, runs north–northwest, and is surrounded by three- to five-story buildings along each side (Fig. 2). Since a seasonal variation was expected in the performance of the reference station and the AQMs, the interval between the calibration periods (CPs) was set to 4 months. This study used two CPs: (1) from June 10, 2017 to June 22, 2017 (CP1) and (2) from October 19, 2017 to December 4, 2017 (CP2). The sunrise and sunset times during CP1 were approximately 03:19 and 22:24, respectively. During CP2, the sunrise time varied from 07:42 to 08:36, and the sunset time varied from 17:28 to 14:45. The angle of the street, placement of the sensors, and height of the western buildings meant that shadows were cast on the area of the sensors at the end of the day, which was found to affect the measurements (see below). During CP2, data obtained from 2 days (October 28–29, 2017) were removed to avoid synchronization problems between the reference station and the AQMs due to the end of daylight-saving time. The reference data and prescaled data from the AQMs were synchronized and converted to hourly averages in order to reduce fluctuations and to comply with common reporting requirements (e.g., the Swedish EQS; SEPA, 2019). In addition, if a reference data point fell below the reference station’s LOD, that data point and the corresponding synchronous prescaled data point were removed from the datasets (see further discussion on data validation below). The resulting mean hourly concentrations were then used in the calibration procedures and for further analyses.

### Calibration procedures

Three different field calibration procedures were compared. The resulting calibrated datasets defined below will be referred to as *postscaled data*, *bisquare data*, and *orthogonal data*. All calibration procedures are based on a linear transformation of the prescaled data given by the following equation:

1 $${y}_{i}=m{x}_{i}+b$$

where *i* refers to a time slot, *y*_*i*_ is the calibrated estimate of the true concentration, *x*_*i*_ is the prescaled concentration, *m* is the slope, and *b* is the offset.

Field calibrated data provided by the manufacturer of the AQMs will be referred to as *postscaled data*. The company uses three different methods for creating postscaled data from prescaled data of co-located AQMs and reference data (if provided). How the company chooses its method was not disclosed, but in general, the AQMs are primarily calibrated against reference data (if available), secondarily calibrated against a “golden AQM,” and lastly calibrated against the average of all co-located AQMs. The golden AQM method involves selecting one of the AQMs as a “reference station” and calibrating the other AQMs against this golden AQM. The final method for generating postscaled data is to create reference station data from the average of all AQMs and to calibrate the AQMs against this average.

The *bisquare data* were obtained using the bisquare linear fit function in the Curve Fitting Tool provided by MATLAB (v.R2018a). This robust method uses an iteratively reweighted least square algorithm. Its advantage is that outliers are weighted less than data points that are more in agreement with the reference station. The fitting parameters obtained (slope *m* and offset *b*) are then applied to the prescaled data according to Eq. (1).

Orthogonal regression is a commonly used calibration method that has the advantage of taking the accuracy of both the sensor system and the reference station into account, and this method usually performs better than ordinary least squares (Dissanaike & Wang, 2003). The difference is that orthogonal regression minimizes the perpendicular distance between the observations and the fitted line, whereas ordinary least square regression minimizes the vertical (or horizontal) distance (ibid). In this study, orthogonal regression linear fits were calculated using the Orthogonal regression tool in Minitab (v.19.1.1). For each AQM, prescaled data (Y) and the corresponding reference data (X) and the ratios of error variances (Y/X) used were 3.7 for NO_2_, 3.7 for NO, and 15 for PM_10_. The fitting parameters obtained were then applied to Eq. (1). The resulting data are here called *orthogonal data*.

### Performance indicators

In the Data Quality Objective (DQO) of the European Air Quality Directive (EU, 2008), the relative expanded uncertainty (*U*_*r*_) is the metric that determines whether a sensor system (for indicative measurements) meets the DQO when compared to outside reference data. The requirements relevant to this study are that *U*_*r*_ × 100% should be below 25% for NO_2_ and NO_x_ and below 50% for PM_10_ (EU, 2008). According to the DQO (ECWG, 2010), *U*_*r*_ is evaluated by the following equation:

2 $${U}_{r}\left({y}_{i}\right)= \frac{2{\left(\frac{\sum {\left({y}_{i}-{b}_{0}-{b}_{1}{x}_{i}\right)}^{2}}{\left(n-2\right)} - {u}^{2}\left({x}_{i}\right)+({b}_{0}+\left({b}_{1}-1\right){x}_{i}{)}^{2}\right)}^{1/2}}{{y}_{i}}$$

where *b*_*1*_ is the slope and *b*_*o*_ is the offset from an orthogonal regression linear fit, *x*_*i*_ are the reference data points, *y*_*i*_ are the AQM-data points (i.e. prescaled, postscaled, or bisquare data points), and *u* is the uncertainty of the reference station (which is 5.0% for NO and NO_2_ and 0.8% for PM_10_). Note that to calculate *U*_*r*_, an orthogonal regression is performed. Because our datasets referred to as orthogonal data were the orthogonal regression linear fits of prescaled data, *U*_*r*_ values were not calculated for the orthogonal data. Therefore, for each AQM, *U*_*r*_ values were evaluated only for the prescaled, postscaled, and bisquare data corresponding to NO, NO_2_, and PM_10_, respectively.

Root mean square error (RMSE), mean normalized bias (MNB), and mean normalized error (MNE) were calculated according to Eqs. (3–5):

3 $$\mathrm{RMSE}= \sqrt{\frac{1}{n}{\sum }_{i=1}^{n}{({y}_{i}-{x}_{i})}^{2}}$$

4 $$\mathrm{MNB}=\frac1n{\textstyle\sum_{i=1}^n}\frac{Y_i-x_i}{x_i}\times100$$

5 $$\mathrm{MNE}=\frac1n{\textstyle\sum_{i=1}^n}\frac{\left|Y_i-x_i\right|}{x_i}\times100$$

where $n$ is the number of measurements, *y*_*i*_ are the data points (i.e., prescaled, postscaled, bisquare, or orthogonal data), and *x*_*i*_ are the reference data points. By sorting all datasets by the corresponding signal strength in the reference data, the RMSE, MNB, and MNE performances may also be compared based on reference data quartiles. These performance metrics are often used to evaluate signal quality. The advantage (and disadvantage) of the RMSE metric is its sensitivity to outliers, which is caused by summing the squared errors in Eq. 3. The MNE metric is more robust to outliers than RMSE, and as defined in Eq. 5, is the mean absolute relative error. Summing the absolute values has the advantage that negative and positive errors cannot cancel each other out, and both types will add to the sum. In the MNB metric, positive and negative errors may cancel each other. If the MNB is 0, the errors cancel and the signal is unbiased with respect to the reference (i.e., “true” values). Otherwise, a negative or positive MNB means that the signal tends to underestimate or overestimate, respectively. The normalization in MNE and MNB emphasizes the relative errors but renders these metrics more sensitive to signal noise. For all performance indicators presented above, smaller values signify better agreement between the test dataset and the reference data.

An optimal calibration procedure should satisfy the EU DQO, give low errors (low RMSE and MNE), and not introduce unwanted bias (low MNB). In reality, it is a question of judicial appraisal. It is reasonable to choose a biased calibration method that gives the lowest MNE as long as the bias is acceptably low. If the AQMs are to be used for regulatory purposes, then the EU DQQ should be fulfilled. However, a prerequisite for successful calibration is that the AQM raw data are correlated with the reference data.

Linear correlations between the AQM prescaled data and the corresponding reference data were analyzed using the two-tailed Pearson correlation (*r*) in SPSS (v.24.0.0.2). SPSS was also used for calculating *r*^2^ and investigating the Q-Q plots. Cross-correlations were tested using MATLAB (v.R2018a) to investigate possible lags in AQM response times compared to reference station response times.

## Results and discussion

### Low pollutant concentrations are recorded

The average concentrations of NO, NO_2_, and PM_10_ for the two calibration periods (the reference data) are presented in Table 1. The recorded levels for all monitored pollutants in CP2 are higher than those recorded in CP1, and the same trend is reflected in the maximum mean hourly concentrations. The range between the minimum and maximum values compared with the period mean values shows that there are large fluctuations in the mean hourly concentrations. This pattern reflects the diurnal traffic patterns, with much higher pollution loads during weekday rush hours and around midday on weekends than during the other times.

**Table 1:** The reference data NO, NO_2_, and PM_10_ mean period concentrations (µg m^−3^), as well as the minimum and maximum mean hourly concentrations, for the two calibration periods CP1 (June 10, 2017–June 22, 2017) and CP2 (October 19, 2017–December 4, 2017)

	Calibration period 1			Calibration period 2		
	Mean (S.D.)	Min	Max	Mean (S.D.)	Min	Max
NO	11.4 (8.8)	3.4	85.2	26.6 (23.7)	3.9	138.3
NO_2_	15.5 (8.9)	5.3	49.9	23.2 (14.5)	5.2	86.9
PM_10_	9.8 (6.1)	2.2	35.7	10.4 (9.1)	2.1	73.4

**Fig. 3:**
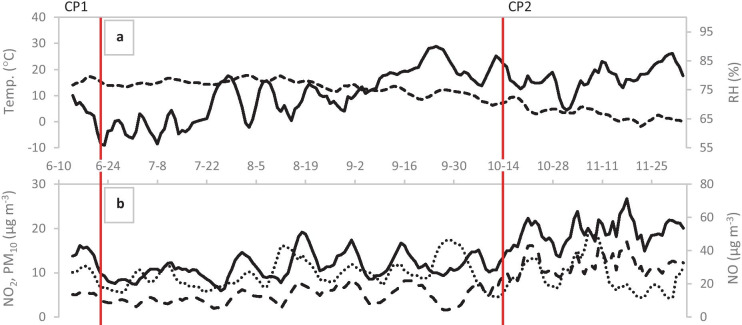
For clarity, 5-day running averages of the mean daily concentrations are presented. **a** Temperature (dashed line) and relative humidity (full line) during the measurement period. **b** Concentrations of NO_2_ (full line), NO (dashed line), and PM_10_ (dotted line) during the measurement period. The two calibration periods are at the beginning and the end of the period shown (CP1: June 10, 2017, to June 22, 2017, end marked with a vertical line; CP2: October 19, 2017, to December 4, 2017, beginning marked with a vertical line)

The large fluctuations in the mean hourly concentrations are smoothed out by looking at averages over longer time periods (running averages). This procedure is necessary for improving the discernibility while showing a longer time series. Figure 3 shows the 5-day running averages of the reference data NO, NO_2_, and PM_10_ mean daily concentrations along with temperatures and relative humidities for the whole measurement period and clearly demonstrates that the pollution loads of NO, NO_2_, and PM_10_ are higher in the autumn (CP2) than in the early summer (CP1). Note that comparatively low levels of the pollutants are recorded for both periods, and that CP2 is roughly five times longer than CP1.

Some trends can be seen when comparing the averages for both time periods to the running averages. For example, the reference data mean period concentrations for NO were, for both CP1 and CP2, below the rural EQS (legal threshold) mean annual threshold: 30 µg m^−3^. In Fig. 3, the 5-day running average of the NO mean daily concentration in CP2 occasionally exceeds 30 µg m^−3^, but the overall average is below this limit. For NO_2_, the mean period concentration for CP1 is lower than the Swedish EQO (goal for 2030) mean annual concentration: 20 µg m^−3^ (SEPA, 2019). It is slightly above this EQO for CP2, but the pooled average for both periods CP1 and CP2 (i.e., 18.7 µg m^−3^) is below the EQO. The NO_2_ reference data during CP2 were occasionally higher than the Swedish EQO mean hourly concentration: 60 µg m^−3^ (SEPA, 2019). During CP2, the latter was exceeded only 23 h or 2.7% of the total measurement time. During CP1, this EQO was not exceeded at any time. The NO_2_ reference data never exceed the Swedish EQS thresholds: 90 µg m^−3^ for the mean hourly or 60 µg m^−3^ for the mean daily concentration (Swedish government, 2019). For PM_10_, both the maximum values for the mean daily concentration (during CP1: 14.5 µg m^−3^ and CP2: 24.3 µg m^−3^, not shown in Table 1) were clearly below the Swedish EQO mean daily concentration (30 µg m^−3^). In spite of the fact that the measurements are conducted beside one of the busier streets in Gävle, the recorded pollutant loads are sufficiently low to already satisfy the EQO goals in most cases.

Measuring such low concentrations of pollutants is a challenge for the reference station and even more for the AQMs. For NO_2_, 16.9% and 15.3% of the raw reference data fall below the reference station LOD in CP1 and CP2 respectively. The corresponding numbers for the raw NO reference data are 26.4% and 18.9% and for the raw PM_10_ reference data, 4.9% and 1.1%. The corresponding AQM LOD statistics based on the reference data during CP1 and CP2 were: NO_2_, 77.4% and 57.3%; NO, 47.5% and 29.5%; and PM_10_, 99.2% and 95.1%. As described above, all data points below the LOD for the reference station were removed from the raw reference data together with the synchronous AQM prescaled data points, and the resulting cleaned datasets will be referred to as reference data and prescaled data below. After data cleaning, most of the reference data for NO, and to a lesser extent the reference data for NO_2_, will be above the AQM LOD. These conclusions are based on the reference data, but since the goal for the prescaled data is to estimate the reference data, the same conclusions are also expected to hold for the prescaled data. For PM_10_, most reference data will still be below the AQM LOD, but this is a likely outcome for measurements in any small city in Sweden, as will be demonstrated by this study. Cleaning the raw datasets in the manner described ensures that the calibration procedures are performed against the best possible reference data.

### Pearson’s correlation between reference data and prescaled data and other statistical tests

The Pearson correlation coefficient (*r*) and the more commonly reported *r*^2^ were evaluated for each AQM prescaled dataset in relation to the reference data. Data cleaning did not change the correlation coefficients appreciably. Note that the data from any linear calibration of the prescaled data will have the same correlation coefficient as the prescaled data. All Pearson’s correlation coefficients evaluated were statistically significant (*p* < 0.01). For all AQM NO prescaled data, evaluated correlation coefficients in CP1 and CP2 were 0.6–0.93 and 0.97–0.99, respectively. The corresponding correlation coefficients for NO_2_ were 0.68–0.85 and 0.86–0.94, and for PM_10_, 0.35–0.58 and 0.32–0.56, respectively. As expected from the number of data points higher than AQM LODs in the prescaled data, the prescaled data for both NO and NO_2_ showed stronger correlations between AQMs and the reference data than the PM_10_ prescaled data. Nevertheless, the *r*^2^ values reported in this study are comparable to those reported in other studies for AQMesh and other low-cost sensors (see Table 2).

**Table 2:** The square of the correlation coefficients (*r*^2^) when NO, NO_2_, and PM_10_ measurements obtained using different low-cost AQMs are compared with data from reference stations (see table legend for references)

Sensor platform	*r*^2^ (NO)	*r*^2^ (NO_2_)	*r*^2^ (PM_10_)
SNAQ^a^	0.34	0.84	0.12–0.15
E-mote^c^	0.25–0.51	0.64	
ECN Airbox^a^		0.89	0.33–0.36
NanoEnvi sensor^a^		0.57	
ENEA Air-Sensors^a^		0.06	0.33
AuTh-ISAG AQ Microsensor box^a^		0.02	
AQMesh^a,b^	0.18–0.93	0.56–0.89	
AQMesh^d^	0.36–0.98	0.46–0.89	0.12–0.34

^a^Borrego et al. (2016)

^b^Cordero et al. (2018)

^c^Munir et al. (2019)

^d^This study

Testing for cross-correlations yielded no apparent time lag in the prescaled data from the AQMs compared to the reference data. Since the reference and prescaled data are attempting to estimate the same underlying variable, the difference between them should ideally be normally distributed. However, such a distribution was not observed for most AQMs in this study. Using the Shapiro-Wilk’s test for normality in the differences, most values fall below the acceptance limit (0.05). Qualitative observations of the SPSS Q-Q plots revealed that deviations from normality mostly occur in the low and/or high end of the concentration range. One interpretation of the deviations from normality is that the prescaled data include outliers, which was one of the reasons that we chose the more robust bisquare fitting method as one of the calibration procedures to be tested.

### Comparisons of *r*^2^ with other air quality sensor platforms

Most studies report the square of the correlation coefficients (*r*^2^), and it is convenient to use this metric to compare studies with each other. Studies using other types of AQMs to measure NO (see Table 2) have reported low correlations between AQM data and reference data. For instance, Borrego et al. (2016) evaluated an *r*^2^ value of 0.34 when comparing another type of AQM (SNAQ) data with reference data. The SNAQ AQM used in that study has the same type of Alphasense sensors as the AQMesh AQM used in this study. Munir et al. (2019) compared the mean of the NO data obtained from ten E-MOTE AQMs around the campus of the University of Sheffield with the NO data obtained from a reference station in the city, but not co-located with the sensors. That study reported an *r*^2^ value of 0.25 after a linear regression fit and showed that this value could be improved to 0.51 by performing multiple linear regression fits using the variables NO and NO_2_ concentrations, wind speed, relative humidity, and temperature.

The *r*^2^ values of NO_2_ measurements varied considerably among the different studies (Table 2). Borrego et al. (2016) reported high *r*^2^ values for the SNAQ and ECN Airbox AQMs, but relatively low *r*^2^ values were reported for the NanoEnvi AQM and very low *r*^2^ values for the AuTh-ISAG and ENEA AQMs. Munir et al. (2019) reported a very low *r*^2^ value of 0.15 for the E-MOTE AQM when fitted using linear regression; they found that the *r*^2^ value could be improved to 0.65 when the data were fitted using a multiple linear regression model. In the present study, comparatively high *r*^2^ values were observed for the AQM NO_2_ and NO data.

For PM_10_ data from low-cost AQMs, studies using optical particle counters demonstrate rather poor correlations with reference data (see Table 2). Borrego et al. (2016) reported *r*^2^ values of 0.33–0.36 for the ECN Airbox and ENEA Air AQMs, but they observed even lower correlations (0.12–0.15) for the SNAQ AQM. In this study, the PM_10_ *r*^2^ values for the AQMs and reference data were in the range of 0.11–0.41, with a mean *r*^2^ value 0.22 and standard deviation of ± 0.07. During CP1 and CP2, even the TEOM 1400ab particle sensor in the reference station occasionally was observed to perform poorly, reporting unphysical negative values. The latter results were naturally removed by the data cleaning. Overall, the low correlation coefficients for the PM_10_ data observed in this study are comparable to those observed in other studies.

### Calibration performance indicators

While the Pearson correlations were not appreciably improved by the data cleaning, that procedure greatly improved the performance indicators RMSE, MNE, MNB, and *U*_*r*_. Note that data cleaning is based on the LODs of the reference station and that AQM data below AQM LODs may still remain in the datasets. The performance indicators obtained from the prescaled data as well as from the calibrated postscaled data, bisquare data, and orthogonal data are each discussed in the following sections. Aggregating the results for all AQMs according to each performance indicator (except *U*_*r*_) into a single boxplot simplifies the presentation of results, but still, 18 figures are required (all of which are presented in the Supplementary material). The *U*_*r*_ calculations (that generate a separate figure for each AQM, dataset, CP, and pollutant) would theoretically yield 144 figures, although some cases were omitted due to, e.g., AQM failures (all *U*_*r*_ figures are presented in the Supplementary material). In the following discussion, a selected sample figure is composed for each of the performance indicators. For each pollutant considered, the most informative figure for RMSE, MNE, or MNB was selected together with two of the *U*_*r*_ plots. Trends in the relative performances of the calibration procedures are relatively easy to discern in this study. Calibration generally improves the performance indicators, and the relative improvements between the calibration procedures basically follow the same trends for all of the performance indicators.

### Nitrogen dioxide

**Fig. 4:**
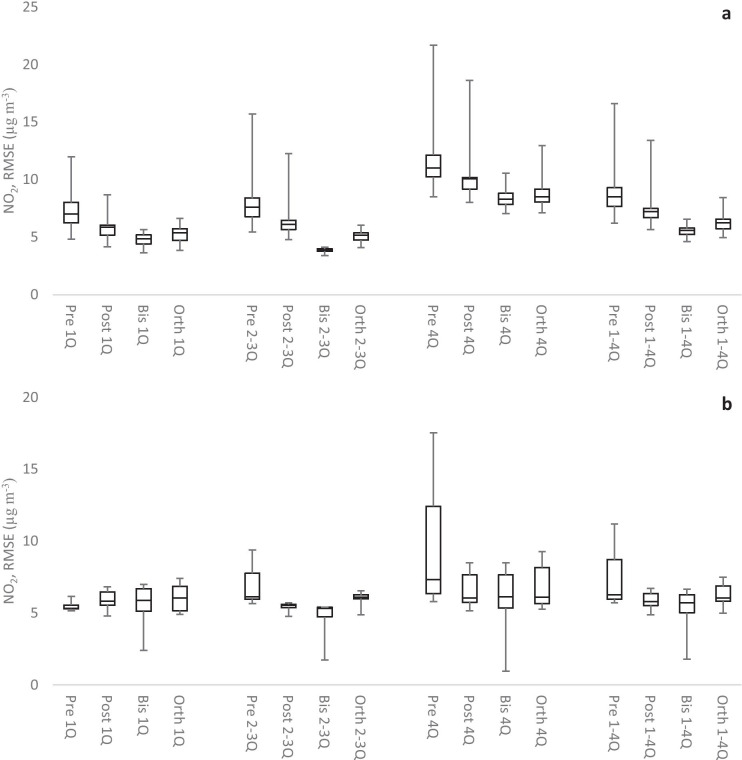
RMSE for NO_2_ during CP1 (**a**) and CP2 (**b**). Boxplot of all AQMs (8 AQMs during CP1 and 6 AQMs during CP2) divided into quartiles. 1Q is the first, 2–3Q is the second and third, 4Q is the fourth quartile, and 1–4Q is the entire data. The whiskers show the min-max, the middle line is the median, and the tops and bottoms of the boxes are the first and third quartiles. Pre, Post, Bis, and Orth are prescaled, postscaled, bisquare, and orthogonal data, respectively

As noted above, the concentrations of NO_2_ are slightly higher in the autumn (CP2) than in the early summer (CP1). Measurements when reference data fell below the AQM LOD also occurred more frequently during CP1 than during CP2. These factors contribute to the observed higher Pearson correlation coefficients for CP2. Similarly, the NO_2_ RMSEs were generally smaller in CP2 as compared with CP1. Figure 4 shows boxplots summarizing the NO_2_ RMSE results of all AQMs and for each dataset, i.e., prescaled, postscaled, bisquare, and orthogonal data, with respect to the reference data. Each boxplot summarizes the following statistics of the RMSEs for all active AQMs: minimum, first quartile, median, fourth quartile, and maximum. Note that RMSEs were also calculated according to quartiles to highlight differences in performance that depend on the level of concentration measured. The RMSE results for the four datasets are grouped (from left to right) into the first quartile (1Q), middle quartiles (2-3Q), and fourth quartile (4Q) with respect to the reference data. Performance boxplots for the entire datasets (including all quartiles, 1-4Q) are presented at the far right-hand side in Fig. 4. In CP1, one AQM performed much worse than the other AQMs in the prescaled data. This was remedied by calibration in the bisquare and orthogonal data, but only partly in the postscaled data. The higher frequency of low concentrations during CP1 appears to have resulted in slightly higher median RMSEs for the 4Q than for the other quartiles. For CP2 (Fig. 4b), the median RMSEs were more uniform over the quartiles but were still slightly higher in 4Q. The ranges of AQM RMSEs, both in terms of box size and max–min, were larger for the 4Q in both CP1 and CP2 than for the other quartiles. Postscaled data only slightly reduced the ranges of RMSEs compared to prescaled data. Orthogonal data reduce the ranges more, and, in CP1, bisquare data reduce the ranges even more. In the CP2 bisquare data, one AQM performed appreciably better than the other AQMs. Overall, the improvement of the median RMSEs was small, but performances increased in the order prescaled, postscaled, orthogonal, bisquare data (ranges and medians of RMSE decreased in the order pre > post > orth > bis).

NO_2_ MNEs were lowest for the 4Q and increased for the other quartiles (compare with Fig. 7, for NO MNEs, to see the general trends). The medians for the NO_2_ MNEs for the entire datasets (1–4Q) range from 30 to 55%. The performances increased, and ranges and medians of MNE decreased in the order pre > post > orth > bis.

The medians for the NO_2_ MNBs for the entire datasets (1–4Q) ranged from −30% to + 10%. The orthogonal data were the least biased with only + 6% and + 4% in CP1 and CP2, respectively. Postscaled data performed slightly better than bisquare data in CP2, but the situation was reversed in CP1. NO_2_ MNBs were predominantly negative in all quartiles for prescaled and postscaled data in CP1 and for prescaled data in CP2. For the orthogonal and bisquare data (and also for the postscaled data in CP2), the NO_2_ MNBs went from positive values in the 1Q to negative in the 4Q. The performances increased and ranges and medians of MNB decreased in the order pre > post ≈ bis > orth.

**Fig. 5:**
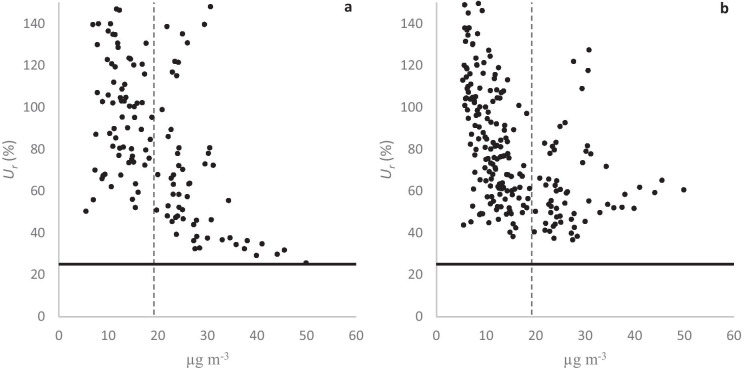
The relative expanded uncertainty (*U*_*r*_) for NO_2_ for postscaled (**a**) and bisquare data (**b**) from AQM Id. no. 734150 during CP1. The solid line is the DQO, and the dashed line is the low-cost AQM’s LOD

*U*_*r*_ may be the most important performance indicator because the EU DQO requires it to be less than 25% for indicative measurements of NO_2_ for regulatory purposes. In CP1, when approximately half of the AQM NO_2_ data points were below the AQM LOD, *U*_*r*_ rarely fell below 25%. While both postscaled and bisquare data substantially reduced the range of *U*_*r*_, compared to prescaled data, this improvement was generally greater for bisquare data. On the other hand, postscaled data performed slightly better than bisquare data at higher concentrations. In CP2, when approximately a third of the AQM NO_2_ data points were below the AQM LOD, the improvement of field calibration was less evident but followed the same general trend. Above 50 µg m^−3^, most *U*_*r*_-plots for both postscaled and bisquare data (and sometimes even prescaled data) satisfied the DQO. Figure 5 shows NO_2_ *U*_*r*_ plots for postscaled and bisquare data from one representative AQM in CP1 (which were generally less satisfactory than in CP2). All NO_2_ *U*_*r*_ plots are supplied in the Supplementary material. In this study, none of the AQM NO_2_ datasets in CP1 satisfied the DQO *U*_*r*_ threshold; however, most datasets in CP2 did. Calibration improved the *U*_*r*_ values with respect to those for the prescaled data in most cases. The postscaled data and the bisquare data performed equally well.

Both the lower correlation and higher number of data points below AQM LOD probably contributed to the failure to satisfy the DQO in CP1. However, during CP2, most of the NO_2_ postscaled and bisquare data satisfied the DQO above 50 µg m^−3^. To summarize the NO_2_ measurements, the performances of all three calibration procedures were roughly equal, and all calibration procedures generally improved performances with respect to the prescaled data.

**Fig. 6:**
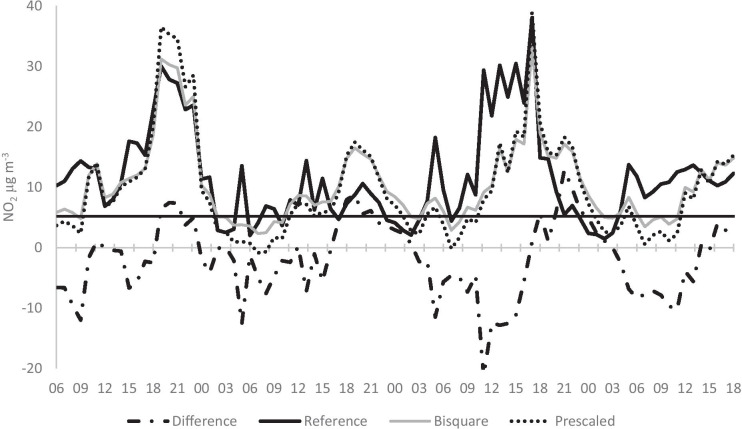
Representative portion of NO_2_ measurements obtained from AQM 1776150, and the reference station between June 17, 2017 and June 20, 2017. The solid line is the reference station data, the dashed line is the bisquare fitted data, the dotted line is the prescaled data, and the dashed-dotted line is the difference between the reference station and the bisquare fitted data. The horizontal line marks the LOD for the reference station at 5.185 μg m^−3^. Measurements lower than LOD of the reference station were eliminated from calibrations, although these data points are presented in this figure for clarity

A periodic systematic error pattern that was observed in the measured NO_2_ concentrations for all AQMs is illustrated in Fig. 6. The difference between the reference data and the calibrated bisquare data show that the NO_2_ concentrations were consistently overestimated starting at approximately 18:00 and ending at 03:00 the next day, followed by an interval of underestimation starting at 03:00 and ending at 18:00. We hypothesize that the observed periodic error may be caused by the AQM being exposed to sunlight, as one of the breakpoints (03:00) corresponds to the approximate time of sunrise. The other breakpoint occurs much earlier than the sunset time, which might be because the buildings on the same (west) side of the street shadowed the AQM in the evening (before sunset proper) (see Fig. 2). In the mornings, when the sun rose in the east (i.e., was shining from the other side of the street), the shadowing effect of the east buildings was much smaller. Sunlight increases temperatures in and around the AQMs, and it could also initiate photochemical conversion of NO_2_ to NO. However, the NO AQM data revealed no corresponding periodic pattern (see below), and it is possible that the NO_2_ AQM response simply decreases with increasing ambient temperature.

### Nitrogen oxide

NO measurements have a clear correlation to traffic (e.g., morning, lunchtime, and evening peaks), but no clear correlation with sunlight was observed in the AQM data. As with NO_2_ described above, the concentrations of NO were slightly higher in autumn (CP2) than summer (CP1). Most of the few cases when NO reference data fell below the AQM LOD probably occurred in CP1. Both conditions contribute to the very high correlations (*r*^2^) observed for the NO prescaled data during CP2 (0.96–0.98). Similarly, the NO RMSEs were predominantly smaller in CP2 than in CP1. NO RMSEs were often smaller than the corresponding NO_2_ RMSEs. The performances increased, and ranges and medians of NO RMSEs decreased in the order pre > post > orth > bis. The relative performances of the NO RMSEs were similar to those observed in the NO_2_ RMSEs (Fig. 4).

**Fig. 7:**
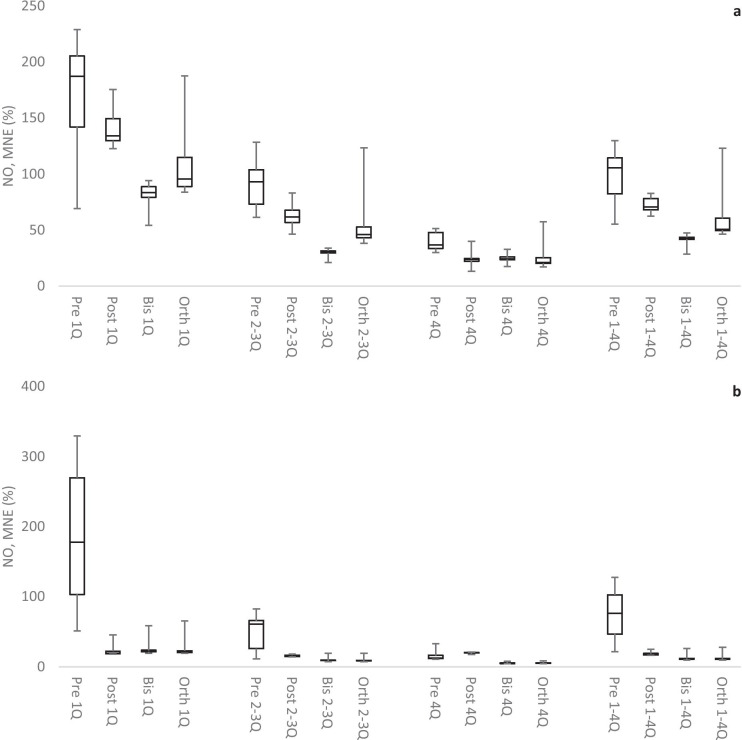
MNE for NO during CP1 (**a**) and CP2 (**b**). Boxplot of all AQMs (8 AQMs during CP1 and 7 AQMs during CP2) divided into quartiles. 1Q is the first, 2–3Q is the second and third, 4Q is the fourth quartile, and 1–4Q is the entire data. The whiskers show the min-max, the middle line is the median, and the tops and bottoms of the boxes are the first and third quartiles. Pre, Post, Bis, and Orth are prescaled, postscaled, bisquare, and orthogonal data, respectively

Figure 7 shows boxplots summarizing the NO MNE results of all AQMs and for each dataset. NO MNEs were lowest for the 4Q and increased in the other quartiles. The median NO MNEs were small (11–18%) in CP2 with the exception of the median NO MNEs for the prescaled data. In CP1, the median NO MNEs were higher and ranged from 43 to 105%. The performances increased, and the ranges and medians of MNE decreased in the order pre > post > orth > bis.

Median NO MNBs for the entire datasets (1–4Q) ranged from −46% to + 48%. The dataset performances increased, and ranges and medians of MNB decreased in the order pre > post > bis > orth. Orthogonal data performed best with median NO MNBs +11% and −1.0% in CP1 and CP2, respectively. The bisquare data median NO MNBs followed closely with +15% and −1.2%, respectively.

**Fig. 8:**
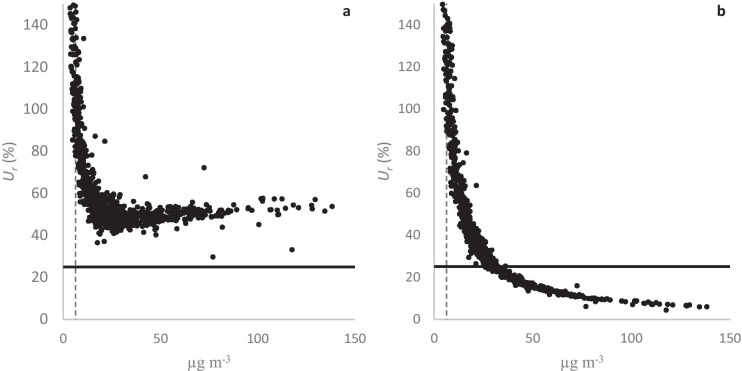
The relative expanded uncertainty (*U*_*r*_) for NO for postscaled (**a**) and bisquare data (**b**) from AQM Id. no. 734150 during CP2. The solid line is the DQO, and the dashed line is the low-cost AQM LOD

*U*_*r*_ is required to be less than 25% for indicative measurements of NO_x_ according to the EU DQO. In the absence of other nitrogen oxides, this DQO should apply to NO. The *U*_*r*_ plots for the datasets rarely satisfied this DQO in CP1. It is unclear whether this was due to AQM sensitivity issues and/or insufficient correlation with NO reference data (0.6–0.93). As observed in the *U*_*r*_ plots in CP1, for NO_2_ (shown above), the bisquare data reduced the range of *U*_*r*_ more than the postscaled data. Postscaled data performed slightly better at higher concentrations. In CP2, prescaled data correlations (*r*) with respect to reference data were higher than 0.97, and the level of NO-concentrations was higher than during CP1. The *U*_*r*_ plots for the bisquare data performed significantly better than the postscaled data, as shown in Fig. 8. All NO *U*_*r*_ plots are supplied in the Supplementary material. At levels of approximately 30 µg m^−3^, all *U*_*r*_ plots for bisquare data satisfied the DQO. None of the *U*_*r*_ plots for postscaled data satisfied the DQO, and postscaled data showed even worse performances than prescaled data.

For the NO measurements, the bisquare data performed as well or better than the other calibration procedures. The orthogonal data performed better than the postscaled data. Only the bisquare data in CP2 satisfied the DQO at levels higher than approximately 30 µg m^−3^.

### Particulate matter

The Pearson correlations and *r*^2^ values for PM_10_ measurements indicated a low linear correlation between the AQMs and reference data during both CPs, and the results revealed no clear difference in *r*^2^ between CP1 and CP2. The lack of correlation may partly be because over 90% of the PM_10_ reference data fell below the AQM LOD, levels at which the AQM’s accuracy may be questionable. Furthermore, many PM_10_ data points were clustered at low values. In the reference data, the median PM_10_ value was approximately 70% of the average value.

The weak correlation was also reflected in the very small reduction in PM_10_ RMSEs for both the postscaled and bisquare data, as compared to prescaled data. The orthogonal data performed even worse than the uncalibrated prescaled data. The performances increased, and ranges and medians of RMSE decreased in the order orth > pre > post > bis. Similar trends were observed for PM_10_ MNEs. Median PM_10_ MNEs for the entire datasets (1–4Q) ranged from 50 to 95%. In CP1, performances increased, and ranges and medians of MNE decreased in the order orth > post > pre > bis. In CP2, median PM_10_ MNEs for the entire datasets (1–4Q) ranged from 39 to 60%, and the performances increased in the order orth > pre > post > bis.

**Fig. 9:**
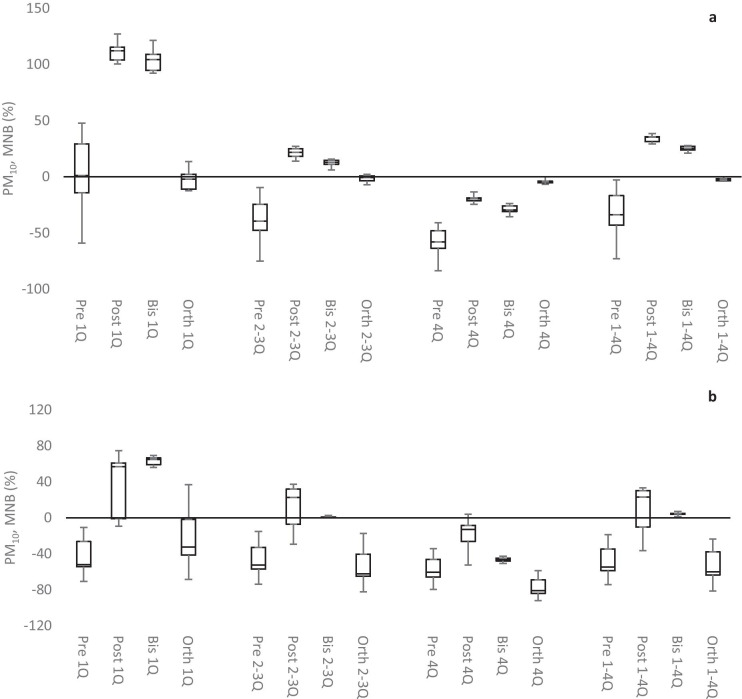
MNB for PM_10_ during CP1 (**a**) and CP2 (**b**). Boxplot of all AQMs (8 AQMs during CP1 and 7 AQMs during CP2) divided into quartiles. 1Q is the first, 2–3Q is the second and third, 4Q is the fourth quartile, and 1–4Q is the entire data. The whiskers show the min-max, the middle line is the median, and the tops and bottoms of the boxes are the first and third quartiles. Pre, Post, Bis, and Orth are prescaled, postscaled, bisquare, and orthogonal data, respectively

Figure 9 shows boxplots summarizing the PM_10_ MNB results of all AQMs and for each data set, i.e., prescaled, postscaled, bisquare, and orthogonal data. The order of performances was different in the different CPs. In CP1, performances increased, and ranges and medians of MNB decreased in the order pre > post > bis > orth. In CP2, the order changed to pre ≈ orth > post > bis.

**Fig. 10:**
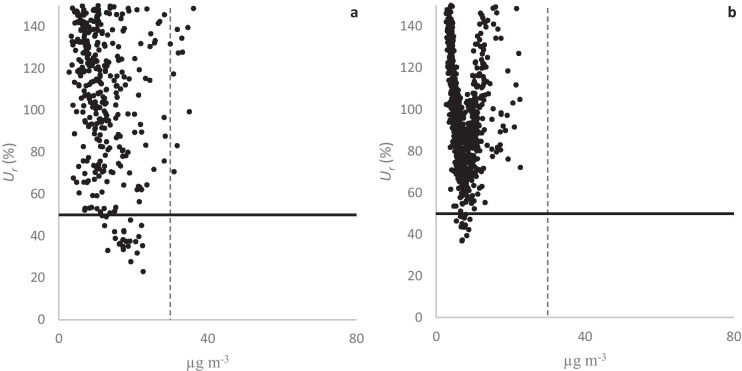
The relative expanded uncertainty (*U*_*r*_) for PM_10_ for postscaled (**a**) and bisquare data (**b**) from AQM Id. no. 707150 during CP2. The solid line is the DQO, and the dashed line is the low-cost AQM LOD

The EU DQO requires *U*_*r*_ to be less than 50% for indicative measurements of PM_10_. Only a few data points in the *U*_*r*_ plots for CP1 and CP2 satisfy this DQO, as may be expected if and when most PM_10_ data points are below the AQM’s LOD. However, as observed for NO_2_ and NO, bisquare data suppressed the ranges of *U*_*r*_ between the AQMs more than postscaled data. Figure 10 shows PM_10_ *U*_*r*_ plots for postscaled and bisquare data from one representative sensor in CP2. For this particular AQM, the percentages of *U*_*r*_ × 100% data points below 150% were 34% and 75% for postscaled and bisquare data, respectively. The data points in Fig. 10 are in a V-shape, with bisquare data *U*_*r*_ performing worse than postscaled data at higher concentrations. This anomaly probably arises from fitting the AQM data with a very low linear correlation to the PM_10_ reference data. All PM_10_ *U*_*r*_ plots are in the Supplementary material.

In this study, very low PM_10_ concentrations were recorded. As noted previously, this situation will probably be encountered in many field studies in smaller cities. Data cleaning by removing the data points below the reference station PM_10_ LOD and the corresponding synchronous AQM data points improves the performances of the calibration procedures in terms of RMSE, MNE, MNB, and *U*_*r*_. It is therefore important to calibrate against the best possible reference data. As a consequence of the low values recorded, performances of the calibration procedures for PM_10_ concentrations above the AQM LOD could not be evaluated in this study. However, for data points below the AQM’s LOD, the MNEs are still less than 50% after calibration which means that the AQM PM_10_ data points below the AQM’s LOD can still be interpreted as (almost) indicative of low PM_10_ concentrations, and these data points need not be removed.

### Hardware reliability and data transfer

For the most part, the functionality of the optical particle counters in the AQMesh AQMs for PM_10_ measurement remained functional for the entire 7-month measurement period of this study, the exception being a single AQM. One AQM’s (Id. no. 693150) counter had a systematic error in which every 16th measurement (15-min interval) failed for an unknown reason, and these data were therefore simply treated as missing. The NO_2_ sensors failed in three AQMs (Id. nos. 788150, 734150, and 706150). None of these sensors were replaced before CP2, and their failures occurred between October 4 and 10, 2017, a period with a considerable amount of rainfall and fluctuating temperatures. Humidity accumulation in the chemical sensors will eventually make them “burst” and leak electrolytes (Alphasense, 2013). The relative humidity was indeed high during the autumn of the measurement period in this study, thus possibly explaining the malfunction of the sensors. Two NO sensors failed: AQM 845150 failed on September 12, and this sensor was replaced at the beginning of CP2; AQM 788150 failed on December 30 and was not replaced. Seven AQMs were powered by batteries, and their expected battery lifetimes (nominally seven months) were exceeded by 7 to 31 days. One AQM (1776150) was powered by a small solar panel. This solar panel did not deliver the required voltage to the AQM when the surrounding buildings at the location of the reference station blocked the sun. Transmissions from this AQM ended in the time between CP1 and CP2.

Data were downloaded from the air sensor database at least once a week during the 7-month measurement period. A problem with data downloading was that only a few days’ worth of NO, NO_2_, and PM_10_ data could be downloaded at a time.

### Evaluation of the AQM sensors and the calibration procedures

Chemical sensors in AQMs deteriorate over time, leading to loss of sensitivity and a decrease in measurement accuracy (Wei et al., 2018). In this study, the AQMs were brand new with intact sensors during CP1. At the start of CP2, the AQMs had been in use outdoors for more than four consecutive months. We therefore expected better sensor performances in CP1 than in CP2, but the opposite was observed—the observed AQM performances were better for CP2 than for CP1. This observation can most probably be explained by the fact that the mean concentrations of NO_2_, NO, and PM_10_ were lower in summer (CP1) than in autumn (CP2). Increases in the AQM uncertainty of measurements at lower concentrations are probably the reason for the lower *r*^2^ values as well the higher values for the performance indicators seen in CP1 compared to CP2. Previous research using AQMesh AQMs also reported that lower concentrations affected the uncertainty of measurements and had a drop in *r*^2^ values during summer (Castell et al., 2016), which is corroborated by the results of this study. Castell et al. (2016) speculated that the reason for the seasonal drop in correlation could be that *r*^2^ varies with the air composition, meteorological conditions, and biases due to sensor detection limits; this study suggests that the latter factor may be the most important.

This work shows that AQM prescaled data need to be calibrated in field conditions to yield more reliable results. In the words of Cross et al. (2017), “it cannot be overstated that EC-sensor systems … can return reliable data only if calibrated over the full range of pollutant concentrations and meteorological parameters that will be encountered when they are deployed.” In this study, three different calibration procedures were compared, i.e., postscaled, bisquare, and orthogonal data. All three calibration procedures reduced the magnitudes, and the ranges of the performance indicators RMSE, MNE, MNB, and gave more reliable results as compared to the prescaled data. The performances in general increased in the order postscaled, orthogonal, and bisquare data. Bisquare data introduced a very slight bias (shown by the MNBs) as compared to orthogonal data but performed better for RMSEs and MNEs. In terms of *U*_*r*_, bisquare data reduced the range of *U*_*r*_ better than postscaled data. Thus, bisquare data performed better than both postscaled data and orthogonal data in most cases.

The low concentrations encountered at field conditions in this study pose the greatest challenge for the AQMs, but also for the linear calibration procedures. When many AQM data points are below the AQM’s LODs, the relative uncertainties of the AQM measurements increase, and the linear correlations with the reference data deteriorate. As a consequence, performance indicators of the calibration procedures increase, as observed in this study. Imposing a linear fit on badly correlated data may also lead to unphysical anomalies, such as the V-shaped *U*_*r*_ clusters observed for the PM_10_ bisquare data (e.g., see Fig. 10). Even though most PM_10_ data points were below the estimated AQM’s LOD, we found the relative errors (MNEs) to be on the order of 50% after calibration, which means that calibrated data below the LOD, for this AQM, may almost be interpreted as indicative.

### Comparison with other AQMesh studies

Several studies using AQMesh have been published recently (e.g., Borrego et al., 2016; Castell et al., 2016, 2018; De Vito et al., 2020; Hickman et al., 2017; Ottosen & Kumar, 2019; Topalović et al., 2019). Cordero et al. (2018) obtained measurements from four AQMesh AQMs placed in two different locations that had been calibrated against reference stations. For NO_2_ measurements, they reported squared correlation coefficients (*r*^2^) between prescaled and reference data on the order of 0.76 ± 0.13 (mean ± standard deviation [S.D.]), which is slightly higher than the *r*^2^ values for NO_2_ obtained in the present study (0.70 ± 0.13). (As stated above, most studies report *r*^2^, and it is therefore a convenient parameter to use for comparisons.) Castell et al. (2018) measured NO_2_ outside kindergartens in Norway and derived an *r*^2^ value of 0.89, indicating a high correlation between the measured data and data obtained from the reference station. For NO, Cordero et al. (2018) and Borrego et al. (2016) reported *r*^2^ values of 0.70 ± 0.27 and 0.80, respectively, which are equal to or lower than the *r*^2^ values we obtained (0.80 ± 0.19). The accuracy of AQMesh is highly dependent on the measured concentration (Castell et al., 2016), and caution should be exercised in comparing correlations between various studies that have been conducted under different conditions and concentrations.

We were also concerned with comparing RMSEs obtained for different calibration procedures. Cordero et al. (2018) report NO_2_ and NO RMSEs for prescaled data in the range 12.9–6.8 µg m^−3^, which decreased to 7.09–3.36 µg m^−3^ when calibrated using machine learning. For comparison, the NO and NO_2_ RMSEs obtained in this study for prescaled data were 16.2–2.9 µg m^−3^, which decreased to 7.4–1.7 µg m^−3^ for bisquare data. Although drawing detailed conclusions from the comparison of machine learning and the calibration procedures used in this study would be difficult, it appears as though our calibration methods reduce the NO and NO_2_ RMSEs by nearly the same order of magnitude. While other studies favor complex multivariate calibration methods (Cordero et al., 2018; Topalović et al., 2019), the bisquare data used in this study suggest that introducing a slight bias in a linear calibration method may improve the performance indicators by the same amount.

## Conclusions

The prevailing meteorological conditions in Gävle were challenging for the NO and NO_2_ gas sensors in this study, and these chemical sensors failed unusually often during and before our second measurement period (CP2), which was a relatively humid autumn season. In our first measurement period in the early summer (CP1), the linear correlations between AQM data and reference data were lower than they were during autumn. These lower correlations in CP1 may have been caused by different meteorological conditions or (more probably) by the lower concentrations measured. The squared correlation coefficients (*r*^2^) obtained in this study for NO_2_, NO, and PM_10_ are comparable to or better than those obtained by other studies using low-cost AQMs. A small periodic systematic prediction error was observed for the AQM NO_2_ calibrated data with respect to reference data. This error correlated with illumination by sunlight and was probably due to a decrease in the AQM NO_2_ signal as the ambient temperature increased. NO measurements correlated more strongly with higher traffic.

Low concentrations of pollutants, far below the EU EQS and even satisfying the Swedish EQO, were recorded. Measuring these low concentrations was challenging for the AQMs, but also for the reference station. Cleaning the raw data by removing data points in the reference data that were below the reference station’s LODs (and the synchronous data points in the AQM prescaled data) was found to improve the performances of the calibration procedures appreciably. For the AQM NO data, the data cleaning removed most data points below the AQM NO LOD. Leaving the AQM NO_2_ and PM_10_ data that may have been below the AQM’s LODs did not seem to create big problems for the calibrations. For the AQM PM_10_ data, where more than 90% of the data points were projected to be below the AQM PM_10_ LOD, the relative errors (MNEs) after calibration were shown to be on the order of 50%. While the EU DQO in terms of *U*_*r*_ were not satisfied for the AQM PM_10_ in terms of *U*_*r*_, these observations may still be interpreted as representing low PM_10_ concentrations.

Field calibration of the AQMs prescaled data against reference data was necessary. All three calibration procedures used in this study significantly reduced the ranges and magnitudes of the performance indicators (as compared with prescaled data) to yield more reliable results. Lesser improvements of the performance indicators were observed when prescaled and reference data were less strongly linearly correlated. Presenting the performance indicators binned into first quartile (1Q), middle quartiles (2–3Q), and fourth quartile (4Q) of the reference data gave further information about the relative performances of the calibration procedures. Overall, the performance of the postscaled data was roughly equal to that of the orthogonal data. The bisquare data usually performed better than the other two. The bisquare data improved the RMSE by the same amount as other studies using complex multivariate calibration methods. The bisquare data improvements of the performance indicator *U*_*r*_ were sufficient for the AQMs to satisfy the EU DQO for NO_2_ (above 50 µg m^−3^) and NO (above 30 µg m^−3^) during CP2.

## Supplementary information

Below is the link to the electronic supplementary material.

Supplementary file1 (DOCX 4292 KB)

## Data Availability

Raw data will be provided on request.

## Abbreviations

AQM: Air Quality Monitor sensor platform
AQMesh: Brand name for the AQMs used in this study
bis: Bisquared data generated by a bisquare fit of prescaled data to reference data
CP: Calibration periods 1 (CP1) and 2 (CP2)
DQO: Data Quality Objective
EC: Electrochemical (sensor)
EQO: Environmental Quality Objective (environmental goals to be reached 2030)
EQS: Environmental Quality Standard (legal thresholds for air pollutants)
EU: European Union
Id. no.: Identity number (for AQMs)
LOD: Limit of detection
MNB: Mean normalized bias
MNE: Mean normalized error
NO: Nitrogen oxide
NO_2_: Nitrogen dioxide
NO_x_: All types of nitrogen oxides
Orth: Orthogonal data generated by an orthogonal fit of prescaled data to reference data
PM: Particulate matter (PM_10_ has an aerodynamic diameter < 10 μm, PM_2.5_ < 2.5 μm diameter, and PM_1_ <1μm diameter)
Post: Postscaled data fit provided by AQMesh of prescaled data to reference data
Pre: Raw data (i.e., prescaled data) from a factory-calibrated AQMesh
Q: Quartile. First quartile (1Q), middle quartiles (2–3Q), and fourth quartile (4Q)
Q-Q plot: Graphic test for normal distribution of data in SPSS
*r*: Pearson’s correlation coefficient
*r*^2^: The square of the Pearson’s correlation coefficient
RMSE: Root mean square error
S.D.: Standard deviation
SEPA: Swedish Environmental Protection Agency
SLB: Stockholm Air and Noise Analysis (*Stockholm Luft och Bulleranalys*)
SPSS: Originally Statistical Package for the Social Sciences
UK: United Kingdom
*U*_*r*_: Relative extended uncertainty (defined in Eq. 2)
WHO: World Health Organization
