# Supplementary material for: Evaluation of field calibration methods and performance of AQMesh, a low-cost air quality monitor
Source: Environ Monit Assess. 2021 Apr 8;193(5):251. doi: 10.1007/s10661-021-09033-x (PMC8032644; doi:10.1007/s10661-021-09033-x)
Supplement: Supplementary file 1 — Supplementary file1 (DOCX 4292 KB) [file 10661_2021_9033_MOESM1_ESM.docx]

Supplementary material

Evaluation of field calibration methods and performance

of a low-cost air quality monitor – AQMesh

Dan Wahlborg^1^^[[1]](#footnote-1)^*, Mikael Björling^1^, Magnus Mattsson^2^

*^1^ Department of Electrical engineering, Mathematics and Science, Faculty of Engineering and Sustainable Development, University of Gävle, Gävle, Sweden*

*^2^Department of Building, Energy and Environmental Engineering, Faculty of Engineering and Sustainable Development, University of Gävle, Gävle, Sweden*

**Figure S1.** Calculations of RMSE during CP1 (a-c) and CP2 (d-f) for NO_2_, NO and PM_10_. Boxplot of all AQMs divided into quartiles. 1Q is the first, 2-3Q is the second and third, 4Q is the fourth quartile, and 1-4Q is the entire dataset. The whiskers show the min-max, middle line is the median and the top and bottom of the box is the first and third quartile. Pre, Post, Bis and Orth are prescaled, postscaled, bisquare and orthogonal data respectively.


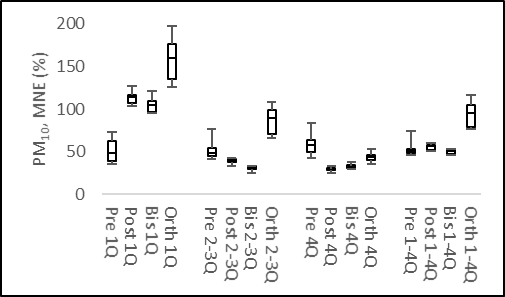


c

a


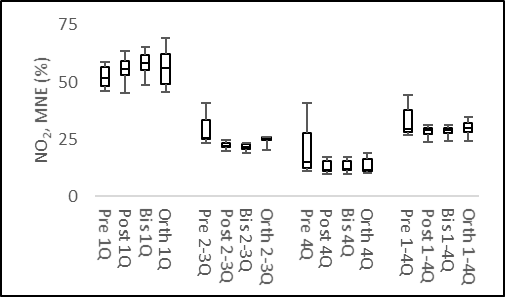


d


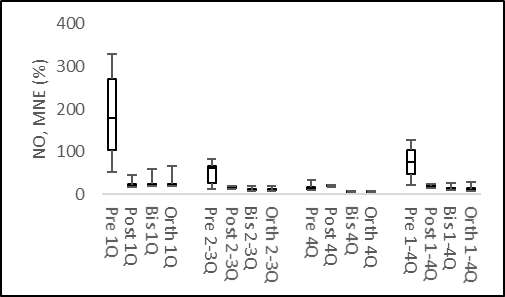


e


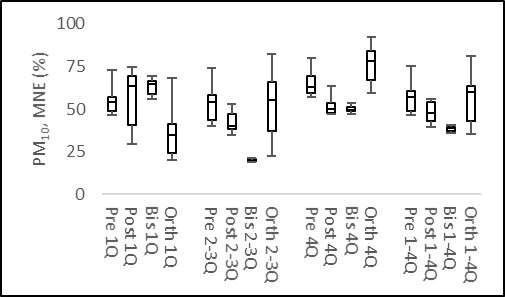


f

**Figure S2.** Calculations of MNE during CP1 (a-c) and CP2 (d-f) for NO_2_ NO and PM_10_. Boxplot of all AQMs divided into quartiles. 1Q is the first, 2-3Q is the second and third, 4Q is the fourth quartile, and 1-4Q is the entire dataset. The whiskers show the min-max, middle line is the median and the top and bottom of the box is the first and third quartile. Pre, Post, Bis and Orth are prescaled, postscaled, bisquare and orthogonal data respectively.

**Figure S3.** Calculations of MNB during CP1 (a-c) and CP2 (d-f) for NO_2_, NO and PM_10_. Boxplot of all AQMs divided into quartiles. 1Q is the first, 2-3Q is the second and third, 4Q is the fourth quartile, and 1-4Q is the entire dataset. The whiskers show the min-max, middle line is the median and the top and bottom of the box is the first and third quartile. Pre, Post, Bis and Orth are prescaled, postscaled, bisquare and orthogonal data respectively.

**Figure S4.** The relative expanded uncertainty (*U_r_*) of NO_2_ measurements of four AQMs (ID 1776150, 801150, 788150 and 845150) during CP1 in relation to measured concentrations made by a reference station. The horizontal line at 25% is the level of acceptable uncertainty for indicative measurements of NO_2_. The vertical dashed line is the LOD for the AQM. Pre, Post, Bis are prescaled, postscaled and bisquare data, respectively.

**Figure S5.** The relative expanded uncertainty (*U_r_*) of NO_2_ measurements of four AQMs (ID 707150, 734150, 693150 and 706150) during CP1 in relation to measured concentrations made by a reference station. The horizontal line at 25% is the level of acceptable uncertainty for indicative measurements of NO_2_. The vertical dashed line is the LOD for the AQM. Pre, Post, Bis are prescaled, postscaled and bisquare data, respectively.

**Figure S6.** The relative expanded uncertainty (*U_r_*) of NO_2_ measurements of four AQMs (ID 801150, 788150, 845150 and 707150) during CP2 in relation to measured concentrations made by a reference station. The horizontal line at 25% is the level of acceptable uncertainty for indicative measurements of NO_2_. The vertical dashed line is the LOD for the AQM. Pre, Post, Bis are prescaled, postscaled and bisquare data, respectively.

**Figure S7.** The relative expanded uncertainty (*U_r_*) of NO_2_ measurements of two AQMs (ID 734150 and 693150) during CP2 in relation to measured concentrations made by a reference station. The horizontal line at 25% is the level of acceptable uncertainty for indicative measurements of NO_2_. The vertical dashed line is the LOD for the AQM. Pre, Post, Bis are prescaled, postscaled and bisquare data, respectively.

**Figure S8.** The relative expanded uncertainty (*U_r_*) of NO measurements of four AQMs (ID 1776150, 801150, 78850 and 845150) during CP1 in relation to measured concentrations made by a reference station. The horizontal line at 25% is the level of acceptable uncertainty for indicative measurements of NO_x_. The vertical dashed line is the LOD for the AQM. Pre, Post, Bis are prescaled, postscaled and bisquare data, respectively.

**Figure S9.** The relative expanded uncertainty (*U_r_*) of NO measurements of four AQMs (ID 707150, 734150, 693150 and 706150) during CP1 in relation to measured concentrations made by a reference station. The horizontal line at 25% is the level of acceptable uncertainty for indicative measurements of NO_x_. The vertical dashed line is the LOD for the AQM. Pre, Post, Bis are prescaled, postscaled and bisquare data, respectively.

**Figure S10.** The relative expanded uncertainty (*U_r_*) of NO measurements of four AQMs (ID 801150, 788150, 845150 and 707150) during CP2 in relation to measured concentrations made by a reference station. The horizontal line at 25% is the level of acceptable uncertainty for indicative measurements of NO_x_. The vertical dashed line is the LOD for the AQM. Pre, Post, Bis are prescaled, postscaled and bisquare data, respectively.

**Figure S11.** The relative expanded uncertainty (*U_r_*) of NO measurements of three AQMs (ID 734150, 693150 and 706150) during CP2 in relation to measured concentrations made by a reference station. The horizontal line at 25% is the level of acceptable uncertainty for indicative measurements of NO_x_. The vertical dashed line is the LOD for the AQM. Pre, Post, Bis are prescaled, postscaled and bisquare data, respectively.

**Figure S12.** The relative expanded uncertainty (*U_r_*) of PM_10_ measurements of four AQMs (ID 1776150, 801150, 788150 and 845150) during CP1 in relation to measured concentrations made by a reference station. The horizontal line at 50% is the level of acceptable uncertainty for indicative measurements of PM_10_. The vertical dashed line is the LOD for the AQM. Pre, Post, Bis are prescaled, postscaled and bisquare data, respectively.

**Figure S13.** The relative expanded uncertainty (*U_r_*) of PM_10_ measurements of four AQMs (ID 707150, 734150, 693150 and 706150) during CP1 in relation to measured concentrations made by a reference station. The horizontal line at 50% is the level of acceptable uncertainty for indicative measurements of PM_10_. The vertical dashed line is the LOD for the AQM. Pre, Post, Bis are prescaled, postscaled and bisquare data, respectively.

**Figure S14.** The relative expanded uncertainty (*U_r_*) of PM_10_ measurements of four AQMs (ID 801150, 788150, 845150 and 707150) during CP2 in relation to measured concentrations made by a reference station. The horizontal line at 50% is the level of acceptable uncertainty for indicative measurements of PM_10_. The vertical dashed line is the LOD for the AQM. Pre, Post, Bis are prescaled, postscaled and bisquare data, respectively.

**Figure S15.** The relative expanded uncertainty (*U_r_*) of PM_10_ measurements of three AQMs (ID 734150, 693150 and 706150) during CP2 in relation to measured concentrations made by a reference station. The horizontal line at 50% is the level of acceptable uncertainty for indicative measurements of PM_10_. The vertical dashed line is the LOD for the AQM. Pre, Post, Bis are prescaled, postscaled and bisquare data, respectively.

1. * Corresponding author. Tel: +46739779308

   E*-mail address*: dan.wahlborg@hig.se [↑](#footnote-ref-1)
